# Neurofeedback as an emerging rehabilitation strategy for Parkinson’s disease: a narrative review

**DOI:** 10.3389/fneur.2026.1866195

**Published:** 2026-09-09

**Authors:** Pierluigi Diotaiuti, Francesco Di Siena, Salvatore Vitiello, Guilherme Vilarino, Anderson D’Oliveira, Alexandro Andrade, Stefania Mancone

**Affiliations:** 1 Department of Human Sciences, Society and Health/University of Cassino and Southern Lazio, Cassino, Italy; 2 Health and Sports Science Center – CEFID/Santa Catarina State University – UDESC, Florianópolis, Brazil

**Keywords:** deep brain stimulation, electroencephalography, functional magnetic resonance imaging, motor symptoms, neurofeedback, neuromodulation, neurorehabilitation, Parkinson’s disease

## Abstract

**Background:**

Parkinson’s disease (PD) is a progressive neurodegenerative disorder characterized by motor and non-motor symptoms associated with dysfunction of cortico-basal ganglia-thalamo-cortical networks. Because current treatments remain largely symptomatic, there is increasing interest in adjunctive rehabilitation strategies capable of targeting disease-relevant neural activity more directly. Neurofeedback (NF), delivered through electroencephalography (EEG), functional magnetic resonance imaging (fMRI), or deep brain stimulation (DBS)-guided platforms, has emerged as a potential self-regulation-based neuromodulatory approach in PD.

**Objective:**

This narrative review aimed to summarize the current empirical evidence on neurofeedback in Parkinson’s disease, with particular attention to neurophysiological feasibility, reported clinical effects, and methodological and translational challenges.

**Methods:**

A narrative review of the literature was conducted using PubMed, Scopus, Web of Science, and Google Scholar. Empirical studies published in English between 2002 and 2026 investigating EEG-based, fMRI-based, or DBS-guided neurofeedback in patients with idiopathic PD were considered. The review qualitatively synthesized findings related to neural target modulation and motor and non-motor outcomes.

**Results:**

Eighteen empirical studies were identified, across EEG-based, fMRI-based, and DBS-guided neurofeedback paradigms. EEG-based protocols represented the largest body of evidence and suggested that at least some patients with PD can achieve partial self-regulation of cortical targets such as sensorimotor rhythms, mu/beta activity, and movement-related cortical potentials. However, associated clinical effects were heterogeneous and generally preliminary. fMRI-based studies supported the feasibility of regulating disease-relevant motor regions and connectivity-based targets, including the supplementary motor area and basal ganglia-related networks, but consistent clinical superiority over active control conditions has not been established. DBS-guided neurofeedback provided the strongest pathophysiological specificity, showing that implanted patients can voluntarily modulate subthalamic beta activity, with preliminary evidence of benefit in selected movement-related outcomes. Across modalities, neurophysiological feasibility appeared more consistently supported than robust clinical efficacy.

**Conclusion:**

Neurofeedback represents a promising and mechanistically grounded adjunctive rehabilitation strategy for Parkinson’s disease. Current evidence indicates that self-regulation of disease-relevant neural signals is feasible in at least a subset of patients, but clinical effectiveness remains uncertain because of small samples, methodological heterogeneity, and limited replication. Future research should prioritize standardized protocols, adequately powered randomized trials, longer follow-up, and better integration of neurofeedback with established pharmacological, rehabilitative, and device-based treatments.

## Graphical abstract

**Figure:**
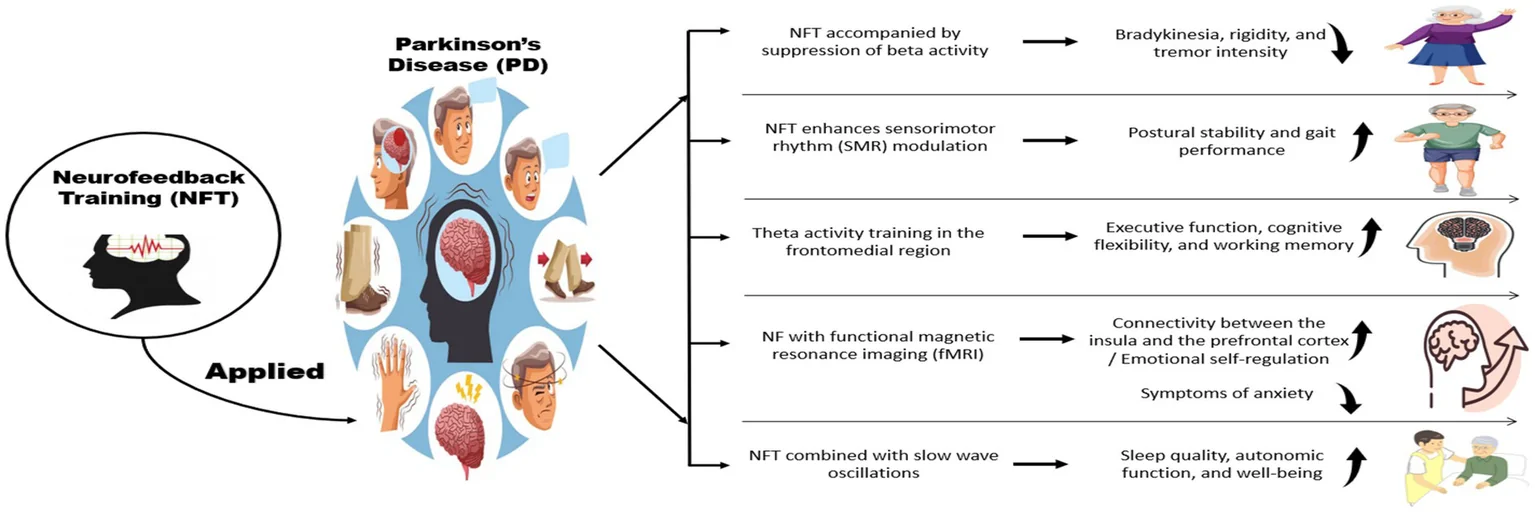

## 1 Introduction

Parkinson’s disease (PD) is a progressive neurodegenerative disorder characterized by degeneration of dopaminergic neurons and widespread dysfunction of cortico-basal ganglia-thalamo-cortical networks. Clinically, PD is defined not only by its cardinal motor manifestations, including bradykinesia, rigidity, resting tremor, and postural instability, but also by a broad spectrum of non-motor symptoms such as cognitive impairment, mood disturbances, sleep disorders, fatigue, autonomic dysfunction, and gastrointestinal symptoms. These non-motor manifestations substantially contribute to disability, reduced quality of life, and caregiver burden, reinforcing the need for therapeutic strategies that go beyond the control of motor symptoms alone (1, 2).

Traditional treatments for PD primarily focus on symptom management rather than halting or reversing disease progression. The cornerstone of PD pharmacological therapy is dopaminergic medication, including levodopa, dopamine agonists, and monoamine oxidase-B inhibitors (MAO-B inhibitors), which temporarily alleviate motor symptoms but become less effective over time due to disease progression and medication-induced complications, such as motor fluctuations and dyskinesia (3).

Beyond pharmacological management, when motor fluctuations and dyskinesia become difficult to control with medication alone, surgical neuromodulation is often considered. In advanced stages of PD, deep brain stimulation (DBS) is often recommended as a neurosurgical intervention targeting the subthalamic nucleus (STN) or globus pallidus interna (GPi) (4). While DBS has demonstrated significant benefits in reducing tremor and rigidity, it is not suitable for all patients, particularly those with cognitive impairments or psychiatric comorbidities (5, 6). Physical rehabilitation and exercise-based therapies have been also recognized as important adjunctive treatments for improving gait, balance, and mobility in PD patients (7–10), these same results have also been recognized in the symptoms of Alzheimer’s disease in comprehensive reviews (11–13). However, these therapies often require continuous engagement, and their long-term benefits are sometimes limited by the progressive nature of PD and patient adherence issues (14).

Current treatments for PD remain predominantly symptomatic. Dopaminergic medications improve many motor symptoms, especially in the earlier stages of disease, but their long-term benefit is often limited by motor fluctuations, dyskinesias, and incomplete efficacy on axial and non-motor symptoms. Rehabilitative interventions and exercise remain important adjuncts, while deep brain stimulation (DBS) is an established option for selected patients with motor complications. However, DBS is invasive, not universally applicable, and does not fully address the multidimensional clinical burden of PD (1, 15). Accordingly, there is growing interest in adjunctive neuromodulatory and neurorehabilitative strategies capable of targeting dysfunctional neural activity more directly and potentially complementing pharmacological and surgical treatments.

Neurofeedback (NF) is one such strategy. By providing real-time information about ongoing neural activity, NF enables individuals to learn voluntary self-regulation of specific brain signals through operant conditioning mechanisms. Depending on the platform used, NF can be delivered through electroencephalography (EEG), real-time functional magnetic resonance imaging (rt-fMRI), or invasive electrophysiological systems integrated with implanted DBS devices. In principle, this approach is particularly attractive in PD because abnormal neural oscillations and dysfunctional network dynamics are central to disease pathophysiology. In particular, excessive beta synchronization within basal ganglia circuits has been consistently associated with bradykinesia and rigidity, and its suppression has been linked to clinical improvement under dopaminergic therapy and DBS, suggesting that disease-relevant neural activity may represent a trainable therapeutic target rather than merely a passive biomarker (16, 17).

Within the broader rehabilitation landscape of PD, NF also aligns with increasing interest in self-regulation-based and motor-imagery-supported interventions. Early rt-fMRI studies demonstrated that patients with PD could learn to upregulate activity within the supplementary motor area during motor imagery, supporting the feasibility of region-specific neurofeedback training in disease-relevant motor networks (18). Subsequent work further suggested that fMRI-based neurofeedback may induce short-term motor improvements and engage compensatory motor circuits, although the clinical magnitude and durability of these effects remain uncertain (19). More recent studies have extended this framework beyond regional activation alone by targeting connectivity-based mechanisms, showing that patients with PD can modulate functionally relevant network coupling during neurofeedback-guided kinesthetic motor imagery (20).

In parallel, EEG-based neurofeedback has emerged as the most accessible and most extensively studied NF modality in PD. A systematic review by Anil et al. concluded that neurofeedback in PD holds promise as a treatment strategy, but also emphasized that the field remains in its infancy, with substantial methodological heterogeneity, limited numerical reporting, and insufficient high-quality randomized controlled trials to establish effectiveness conclusively (16). More recent syntheses have refined this picture, suggesting that while EEG-based neurofeedback can often achieve modulation of the targeted neural signal, evidence for consistent downstream improvement in general motor symptom severity remains mixed and still preliminary (21). This distinction is important, because it suggests that neurophysiological feasibility is increasingly supported, whereas clinical efficacy has not yet been robustly established.

Among newer developments, DBS-guided neurofeedback is particularly noteworthy because it provides direct access to pathological subthalamic activity through fully implanted systems. In a proof-of-concept study, patients with PD learned to voluntarily modulate subthalamic beta oscillations recorded from DBS electrodes, supporting the feasibility of self-regulation at the level of disease-relevant subcortical rhythms (17). More recent work has further suggested that beta-neurofeedback may influence beta-burst dynamics and movement-related performance, strengthening the mechanistic rationale for this approach even though evidence remains limited to small and highly selected patient cohorts (22). These advances indicate that NF in PD is evolving from cortical self-regulation paradigms toward more pathophysiologically specific models of endogenous neuromodulation.

The current literature indicates that neurofeedback is a promising but still developing adjunctive strategy for Parkinson’s disease. Its main appeal lies in its capacity to combine neuromodulation, motor learning, and patient-driven self-regulation within a rehabilitation framework. However, the evidence base remains heterogeneous with respect to modality, neural target, training intensity, study design, and outcome measures. The aim of the present narrative review is therefore to summarize the current empirical evidence on EEG-based, fMRI-based, and DBS-guided neurofeedback in PD, with particular attention to neurophysiological feasibility, reported clinical effects, and the methodological and translational challenges that currently limit routine clinical implementation. Unlike prior syntheses that focused on a single NF platform, such as EEG-only systematic reviews and meta-analyses (16, 21), the present review adopts a cross-modality comparative framework that places EEG-, fMRI-, and DBS-guided neurofeedback side by side and explicitly separates neurophysiological feasibility from clinical efficacy for each platform, an angle not previously addressed jointly in the PD neurofeedback literature.

An updated synthesis is warranted for reasons beyond the simple accumulation of new studies. First, the empirical base has changed substantially since the most recent single-platform reviews: more than a third of the studies included here [e.g., (22–28)] were published after Anil et al. (16), and DBS-guided neurofeedback in particular has only emerged as a distinct research line within this period; a review restricted to EEG alone, or one that predates these studies, cannot capture the field’s current trajectory. Second, and more importantly, considering the three platforms together makes it possible to trace how the field has evolved: from early single-case and small-sample feasibility work establishing that disease-relevant signals could be voluntarily modulated at all [e.g., (18, 29)], through a middle phase of methodologically more structured but still largely uncontrolled EEG- and fMRI-based studies targeting increasingly specific cortical and network-level signals [e.g., (20, 30–32)], to a recent phase combining neurofeedback with other interventions and moving toward subcortical, implant-based precision targeting [e.g., (22, 26, 33)]. Reading the literature along this timeline, rather than only by platform, foregrounds a recurring and clinically consequential pattern that is only partially discussed in prior reviews: neurophysiological feasibility has been demonstrated repeatedly and increasingly precisely, while robust clinical efficacy has not kept pace, and the heterogeneity in protocols, samples, and outcome measures across this 24-year span is itself an obstacle to closing that gap. These two threads (the feasibility-efficacy gap and cross-study heterogeneity) are treated here not as isolated limitations but as organizing themes that run through the Results and Discussion sections below.

## 2 Methods

This review follows a narrative approach to synthesize current evidence on neurofeedback (NF) applications in Parkinson’s disease (PD). The present review followed a previously published narrative review model as a methodological development protocol according to Diotaiuti et al. (34). Because the eligible literature spans heterogeneous, largely non-randomized study designs (case reports, feasibility studies, and small controlled trials), the search strategy and eligibility criteria were structured using the SPIDER framework (Sample, Phenomenon of Interest, Design, Evaluation, Research type) rather than a strictly clinical-trial-oriented framework such as PICO, as SPIDER is better suited to questions combining intervention feasibility with heterogeneous study designs. Specifically: Sample—adults with idiopathic Parkinson’s disease; Phenomenon of Interest—neurofeedback delivered via EEG-, fMRI-, or DBS-guided platforms; Design—empirical studies of any design reporting original data (case reports, feasibility/pilot studies, single-arm or controlled trials); Evaluation—neurophysiological target modulation and PD-relevant motor or non-motor outcomes; Research type—quantitative and mixed-methods empirical research. Reporting of the review methods follows the transparency recommendations for narrative reviews outlined in the SANRA (Scale for the Assessment of Narrative Review Articles) criteria. The bibliographic search and methodological paths followed by the narrative review to achieve the objectives of this study are shown in Table 1.

**Table 1:** Methodological approach for selecting studies that examined the application of NF in the rehabilitation of people with PD.

Element	Description
Databases searched	PubMed, Scopus, Web of Science, and Google Scholar
Keywords used	Main Terms: “Parkinson’s disease and neurofeedback,” “EEG-based neurofeedback in PD,” “fMRI neurofeedback in movement disorders,” “brain oscillations in Parkinson’s,” and “neuroplasticity in PD rehabilitation”
Combination of keywords	Boolean operators (“AND,” “OR”) were used to combine keywords. For example, “Parkinson’s disease” AND Neurofeedback, “EEG-based neurofeedback” AND “Parkinson’s disease”
Bibliographic search period and language	Only articles published in English in the last 25 years (2002–2026)
Inclusion criteria	(1) Investigated EEG-based, fMRI-based, or DBS-guided/invasive neurofeedback interventions in patients with idiopathic Parkinson’s disease; (2) Reported neurophysiological indices of neurofeedback learning and/or PD-relevant motor or non-motor outcomes; (3) Used objective neurophysiological, behavioral, or clinical measures to evaluate neurofeedback-related effects; (4) Included human participants diagnosed with idiopathic Parkinson’s disease.
Exclusion criteria	(1) Lacked sufficient methodological description or did not provide extractable PD-specific findings; (2) Investigated neurofeedback applications in neurological or psychiatric disorders other than Parkinson’s disease without PD-specific outcomes; (3) Were opinion papers, editorials, or non-peer-reviewed sources included only for contextual discussion rather than empirical synthesis.
Data screening and extraction process	The initial screening was based on titles and abstracts, followed by full-text review of potentially relevant articles to assess eligibility. Two authors independently participated in study selection and data extraction, and disagreements were resolved through discussion with a third author. A total of 146 records were identified through database searching. After removal of 38 duplicates, 108 records were screened by title and abstract, of which 72 were excluded as not relevant. The remaining 36 full-text articles were assessed for eligibility, and 18 were excluded (e.g., insufficient methodological description, non-PD-specific outcomes, or non-empirical format), for a total of 90 records discarded during screening and eligibility assessment (see the PRISMA flow diagram in Figure 1). A total of 18 empirical studies met the inclusion criteria and were included in the qualitative synthesis. These comprised 11 EEG-based neurofeedback studies, 4 fMRI-based neurofeedback studies, and 3 DBS-guided/invasive neurofeedback studies.
Measures to improve consistency and minimize bias	The same pre-agreed Boolean search string was run independently in each database by two reviewers to reduce search-execution variability. Title/abstract screening and full-text eligibility assessment were each performed independently by two authors against the pre-specified inclusion/exclusion criteria in this table, with a calibration round on a 10% sample of records before full screening to check agreement on criteria interpretation; all disagreements were resolved by discussion, with a third author adjudicating unresolved cases. Data extraction used a shared, piloted extraction form (study design, sample size, NF modality and target signal, training protocol, outcome measures, and direction of effect) completed independently by two authors and cross-checked for discrepancies. No automated or AI-based tool was used to screen or select studies.
Main outcomes assessed	The main outcomes considered in this review included neurophysiological target modulation, motor performance, and selected non-motor or behavioral outcomes when reported. The studies reviewed encompassed EEG-based neurofeedback targeting beta-band activity, sensorimotor rhythms, or movement-related cortical potentials; fMRI-based neurofeedback targeting motor-region activation or connectivity-based networks; and DBS-guided neurofeedback targeting subthalamic beta activity and related oscillatory dynamics.

**Figure 1:**
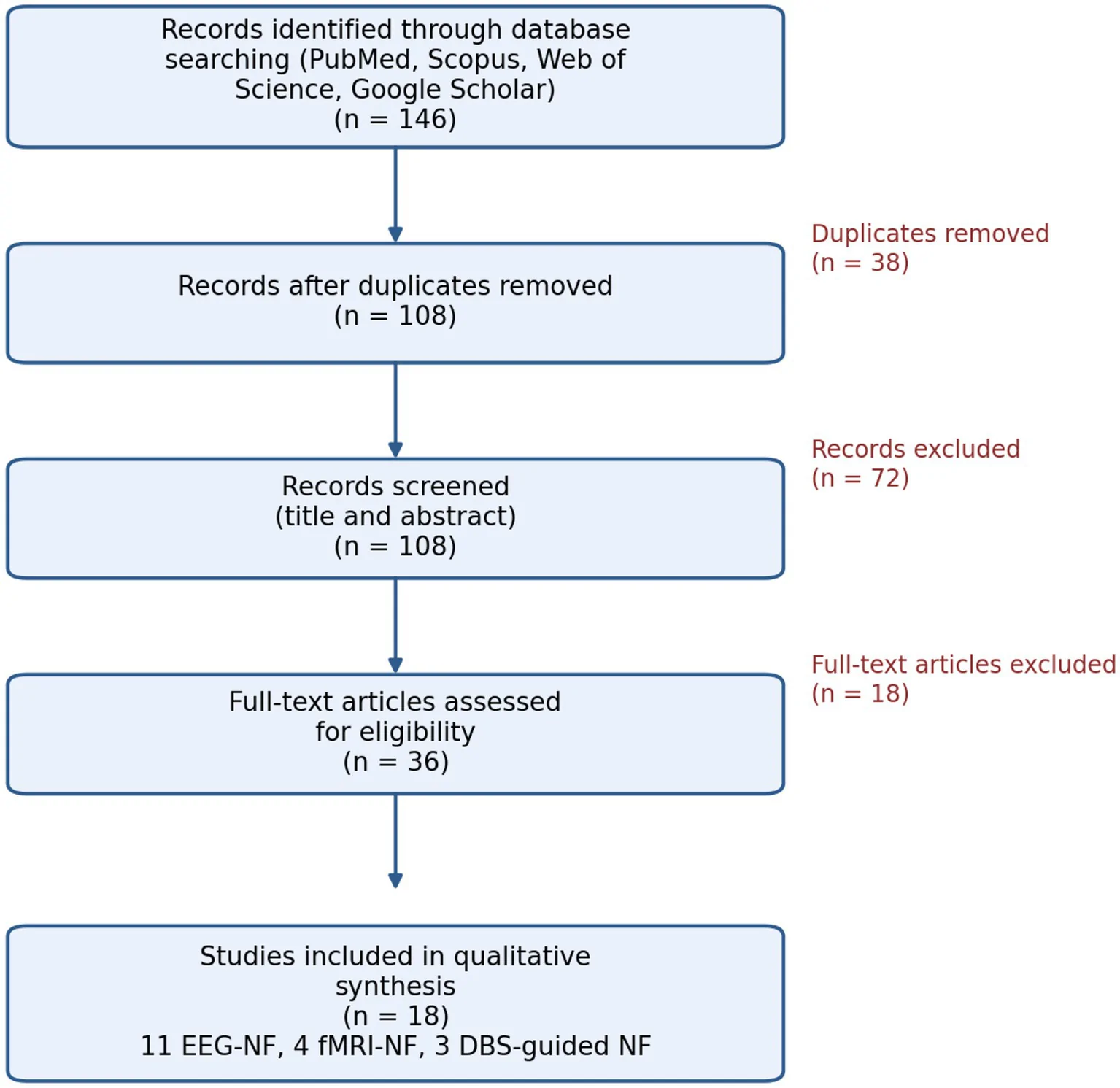
PRISMA flow diagram of study identification, screening, eligibility assessment, and inclusion. Total records discarded during screening and eligibility assessment (excluding duplicates): 72 + 18 = 90.

Figure 1 presents the PRISMA flow diagram summarizing the identification, screening, eligibility assessment, and inclusion of studies for this review.

Given the heterogeneity in study designs, a qualitative synthesis approach was employed to identify trends, gaps, and methodological inconsistencies in the literature. Meta-analytical techniques were not applied due to variability in NF protocols, outcome measures, and participant characteristics across studies. Table 2 summarizes the empirical studies included in the Results section and presents them in chronological order, in order to illustrate the evolution of neurofeedback approaches in Parkinson’s disease from early EEG and fMRI proof-of-concept studies to more recent home-based EEG and DBS-guided paradigms.

**Table 2:** Empirical neurofeedback studies in Parkinson’s disease, ordered chronologically.

Year	Study	Modality	Design/sample	Target/protocol	Main findings
2002	Thompson and Thompson	EEG-NF	Case study, 1 PD patient	Low beta/SMR reinforcement; alpha inhibition	Early proof-of-concept report suggesting feasibility of EEG-based neurofeedback in PD.
2011	Subramanian et al.	fMRI-NF	Proof-of-concept study in PD	SMA upregulation during motor imagery	Patients learned to increase SMA activity, with preliminary short-term motor improvement.
2012	Erickson-Davis et al.	EEG-NF	Pilot study	Alpha reinforcement with theta/high-beta inhibition	Preliminary evidence of symptom-related benefit and good acceptability, but small and exploratory.
2013	Fumuro et al.	EEG-NF	Clinical experimental study	Bereitschaftspotential/readiness-potential augmentation	Suggested that movement-related cortical activity could be voluntarily increased.
2014	Azarpaikan et al.	EEG-NF	Controlled study	Low-beta reinforcement and theta inhibition	Reported improvements in physical balance and postural performance.
2016	Subramanian et al.	fMRI-NF	Phase I randomized trial	fMRI-neurofeedback-guided motor imagery vs. control imagery	Safe and feasible; immediate motor benefit suggested, but superiority over active control remained uncertain.
2018	Kasahara et al.	EEG-NF/BCI	Case report	C3–C4 SMR asymmetry modulation	Demonstrated feasibility of SMR-based self-regulation, with modulation seen especially in the ON-medication condition.
2021	Cook, Pfeifer, & Tass	EEG-NF	Case study	SMR/beta burst-rate modulation over motor cortex	Showed successful cortical self-regulation with exploratory motor benefit.
2022	Legarda et al.	EEG-NF	Case series, 3 PD patients	Infra-low-frequency neuromodulation / neurofeedback	Reported improvements in tremor, writing, and gait/freezing-related symptoms in a nonsurgical setting.
2022	Tinaz et al.	fMRI-NF	Randomized trial	Connectivity-based NF targeting insula–dmFC during kinesthetic motor imagery	Mixed neural and clinical findings; feasibility confirmed, but efficacy remained uncertain.
2023	Han et al.	EEG-NF	RCT	Imagined-movement-based SMR neurofeedback	Supported feasibility of regulating SMR relative power, with exploratory motor and non-motor outcomes.
2023	Shi et al.	EEG / multimodal biofeedback	RCT	Multimodal biofeedback including EEG-SMR regulation	Reported improvements in motor and non-motor outcomes, but the EEG-specific contribution was difficult to isolate.
2024	Cooke et al.	EEG-NF	Home-based within-subject feasibility study	Mu-power reduction with auditory feedback and handgrip task	Demonstrated feasibility and acceptability of at-home EEG neurofeedback; no clear clinical motor differences, but good tolerability.
2024	Romero et al.	EEG-NF	Randomized four-arm controlled trial	EEG-guided NF alone or combined with bilateral high-frequency rTMS	Combined intervention outperformed NF alone on several outcomes; EEG-NF-only effects were more limited.
2024	Rohr-Fukuma et al.	DBS-guided NF	Study in implanted PD patients	Voluntary downregulation of subthalamic beta using a fully implanted DBS system	Patients learned rapid voluntary control of pathological beta activity; median beta reduction reached 31% in the final block.
2025	Baqapuri et al.	fMRI-NF	Repeated-session study in PD	Putamen and SMA as motor neurofeedback targets	Supported feasibility of basal-ganglia-focused neurofeedback, especially putaminal targeting.
2025	Salzmann et al.	DBS-guided NF	Movement-assessment study in implanted PD patients	Short-term beta downregulation with IMU-based motor testing	Reported improvements in selected lower-limb movement-quality metrics after DBS neurofeedback.
2026	Bichsel et al.	DBS-guided NF	Deep-brain recording study in PD	Beta-neurofeedback assessed via beta-burst dynamics	Showed that DBS-based neurofeedback modulated beta-burst characteristics, strengthening evidence for subcortical self-regulation.

## 3 Results

### 3.1 EEG-based neurofeedback studies

Most of the empirical literature on neurofeedback in Parkinson’s disease has focused on EEG-based protocols, making EEG the most extensively investigated modality to date (24–26, 28–32, 35–37). Across the available EEG studies, the most frequently targeted signals include sensorimotor rhythm (SMR), mu/beta-band activity, and movement-related cortical potentials such as the Bereitschaftspotential or readiness potential (24, 25, 31, 32, 35). In terms of the direction of neurophysiological change, most EEG-NF protocols were designed to increase sensorimotor rhythm and low-beta power while simultaneously decreasing high-beta or mu-band activity, in line with the rationale of reducing pathological beta synchronization associated with bradykinesia and rigidity (25, 30, 31, 35). Overall, the EEG literature suggests that at least some patients with PD can achieve partial self-regulation of targeted cortical activity, although the magnitude and clinical relevance of associated behavioral effects remain heterogeneous and generally preliminary (24–26, 30).

The earliest EEG-based studies were case reports or small exploratory investigations primarily aimed at establishing feasibility. These early reports suggested that patients with PD could engage with neurofeedback paradigms and voluntarily modulate selected cortical rhythms under structured feedback conditions (29, 36). Subsequent studies expanded this approach by targeting low-beta reduction, SMR enhancement, or readiness-related cortical activity, with preliminary improvements reported in balance, postural control, and movement preparation (30, 31). However, these findings were generally derived from small samples and heterogeneous protocols, limiting their generalizability.

More recent EEG studies have adopted more structured experimental designs. Controlled and randomized investigations have examined imagined-movement-based SMR training, infra-low-frequency neurofeedback, and multimodal biofeedback interventions incorporating EEG-derived targets (25, 28, 37). Collectively, these studies support the feasibility of EEG-based target modulation, but also suggest that clinical effects may be modest, domain-specific, or difficult to attribute exclusively to the neurofeedback component when multimodal protocols are used (26, 28).

An important translational development is the emergence of home-based EEG neurofeedback. Cooke et al. (24) showed that repeated EEG-NF training could be implemented in domestic settings with acceptable tolerability and adherence, supporting the practical feasibility of portable neurofeedback for self-managed rehabilitation. At the same time, home-based feasibility does not yet resolve the central question of efficacy, since clear motor improvements were not consistently demonstrated (24). Similarly, a four-arm controlled trial by Romero et al. (26) suggested that neurofeedback alone may produce more limited effects than combined approaches, as the combination of EEG-guided NF with bilateral high-frequency rTMS outperformed NF alone on several outcomes.

**Figure 2:**
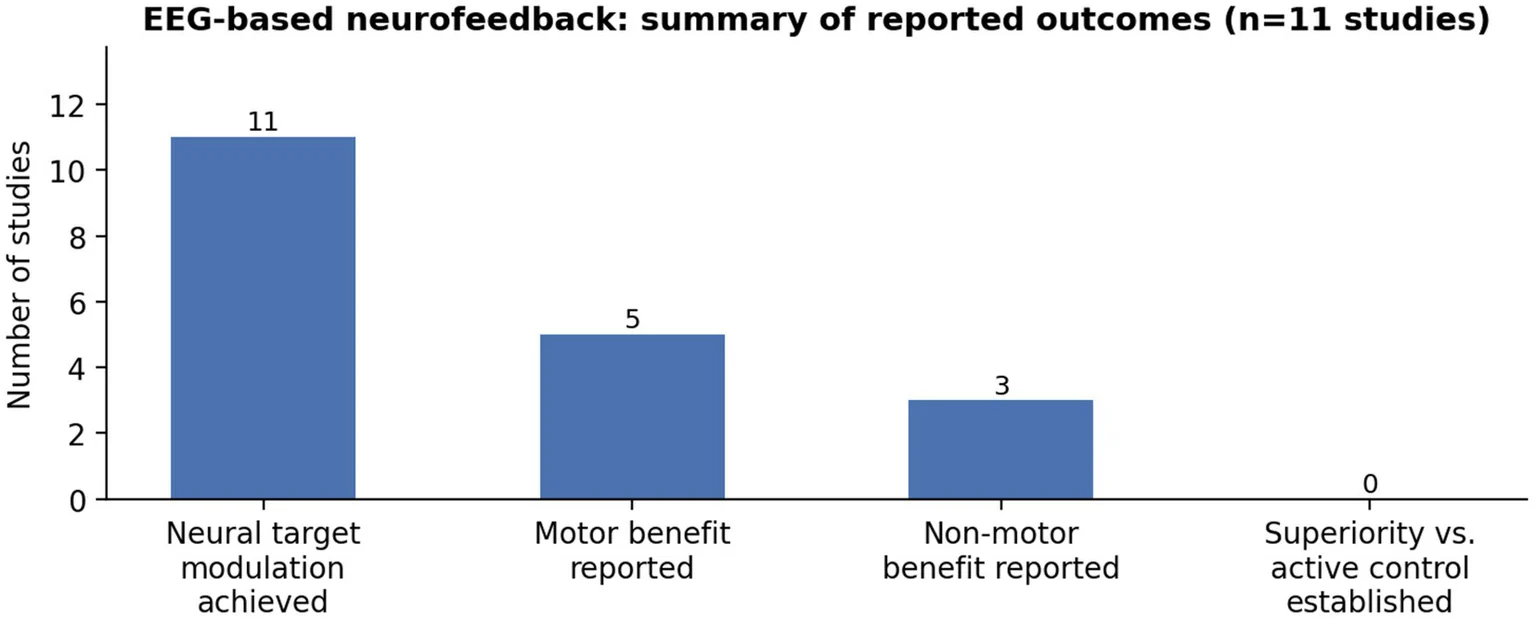
Summary of key EEG-based neurofeedback results extracted from the studies in Table 2.

EEG-based neurofeedback currently appears to be the most mature and accessible NF modality in PD from a practical standpoint, as reflected by the number and variety of studies available (21). However, the evidence base remains characterized by heterogeneous protocols, small samples, and variable outcome measures, and larger randomized studies using harmonized targets and clinically meaningful endpoints are still required to determine whether successful EEG self-regulation translates into reproducible rehabilitation benefit (21, 24–26). Figure 2 summarizes, across the 11 EEG-based neurofeedback studies reviewed above, how many reported successful neural target modulation, motor benefit, non-motor benefit, and superiority over an active control condition.

### 3.2 fMRI-based neurofeedback studies

Compared with EEG-based approaches, the fMRI-neurofeedback literature in Parkinson’s disease is smaller but mechanistically informative (18–20, 23). Early proof-of-concept work demonstrated that patients with PD could increase activation in motor-related cortical regions, particularly the supplementary motor area (SMA), during motor imagery supported by real-time feedback (18). These findings were important because they showed that NF learning in PD was not restricted to surface electrophysiological signals and could also be achieved using region-specific hemodynamic targets. Across fMRI-NF protocols, the trained direction was consistently an upregulation of blood-oxygen-level-dependent (BOLD) activity within the targeted region (e.g., SMA, putamen) or an increase in the strength of targeted functional connectivity, rather than downregulation, reflecting the aim of engaging compensatory or under-recruited motor circuits (18, 20, 23).

Subsequent randomized work confirmed the feasibility and safety of fMRI-neurofeedback in PD, but yielded more mixed clinical results. In particular, Subramanian et al. (19) reported that fMRI-neurofeedback-guided motor imagery was feasible and associated with immediate motor benefit, although superiority over the active control condition remained uncertain. This distinction is important because it suggests that successful regulation of the trained neural target does not automatically produce robust clinical improvement.

More recent fMRI studies have moved beyond cortical motor regions toward network- and basal-ganglia-focused targets. Tinaz et al. (20) examined connectivity-based neurofeedback targeting the insula and dorsomedial frontal cortex during kinesthetic motor imagery, confirming feasibility but reporting mixed neural and clinical effects. Baqapuri et al. (23) further extended the field by investigating the putamen and SMA as motor neurofeedback targets across repeated MRI sessions, supporting the feasibility of basal-ganglia-focused neurofeedback, particularly for putaminal targeting. These studies broaden the conceptual scope of fMRI-neurofeedback in PD by showing that training can be directed not only at cortical activation but also at disease-relevant network and subcortical targets.

**Figure 3:**
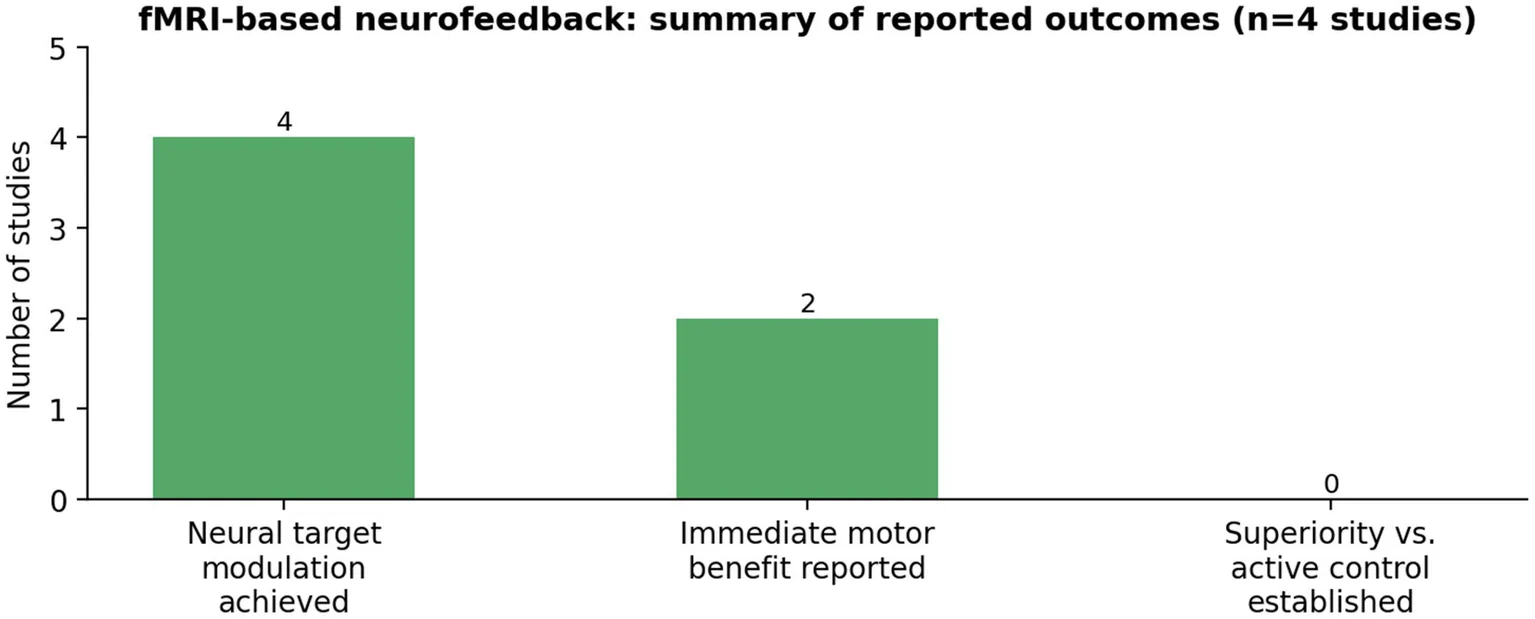
Summary of key fMRI-based neurofeedback results extracted from the studies in Table 2.

The fMRI literature supports the idea that patients with PD can engage in region-specific and connectivity-based neurofeedback involving disease-relevant motor networks (18, 20, 23). However, as with EEG-based approaches, the current evidence remains limited by small samples, variable targets, and insufficient replication. At present, fMRI-NF should therefore be interpreted primarily as a promising mechanistic and translational platform rather than an established clinical intervention (19, 20, 23). Figure 3 summarizes, across the 4 fMRI-based neurofeedback studies reviewed above, how many reported successful neural target modulation, immediate motor benefit, and superiority over an active control condition.

### 3.3 DBS-guided neurofeedback studies

DBS-guided neurofeedback represents one of the most innovative recent developments in the field (22, 27, 33). Unlike EEG- or fMRI-based approaches, DBS-guided paradigms provide access to pathological activity recorded directly from implanted therapeutic devices, particularly subthalamic beta oscillations that are strongly implicated in PD motor dysfunction. This gives DBS-based neurofeedback a particularly strong pathophysiological rationale.

Recent studies using fully implanted systems have shown that patients with PD can achieve rapid voluntary downregulation of subthalamic beta activity during feedback training. Rohr-Fukuma et al. (33) reported that implanted patients were able to learn rapid voluntary control of pathological beta activity using a fully implanted DBS system, with a median beta reduction of 31% in the final training block. This finding is highly relevant because it demonstrates that neurofeedback-based self-regulation can be extended from cortical signals to subcortical disease-relevant oscillations. Unlike EEG- and fMRI-based paradigms, which generally target an increase in the trained signal, DBS-guided protocols were specifically designed to produce a decrease in subthalamic beta power and beta-burst duration, consistent with the well-established inverse relationship between beta activity and motor performance in PD (22, 33).

Subsequent work has suggested that short-term beta downregulation may be associated with improvements in selected movement-quality metrics rather than uniform global motor benefit. In particular, Salzmann et al. (27) reported improvements in selected lower-limb movement-quality measures following DBS neurofeedback, while comparable effects were not observed across all motor domains. This pattern suggests that the clinical effects of subcortical neurofeedback may be domain-specific rather than broadly distributed across motor outcomes.

Another line of DBS-guided work has focused on beta-burst dynamics rather than beta power alone. Bichsel et al. (22) showed that DBS-based neurofeedback modulated beta-burst characteristics in deep brain recordings, strengthening the mechanistic evidence for subcortical self-regulation in PD. Together, these studies support the biological plausibility of DBS-guided NF and indicate that it may directly engage disease-relevant oscillatory mechanisms.

**Figure 4:**
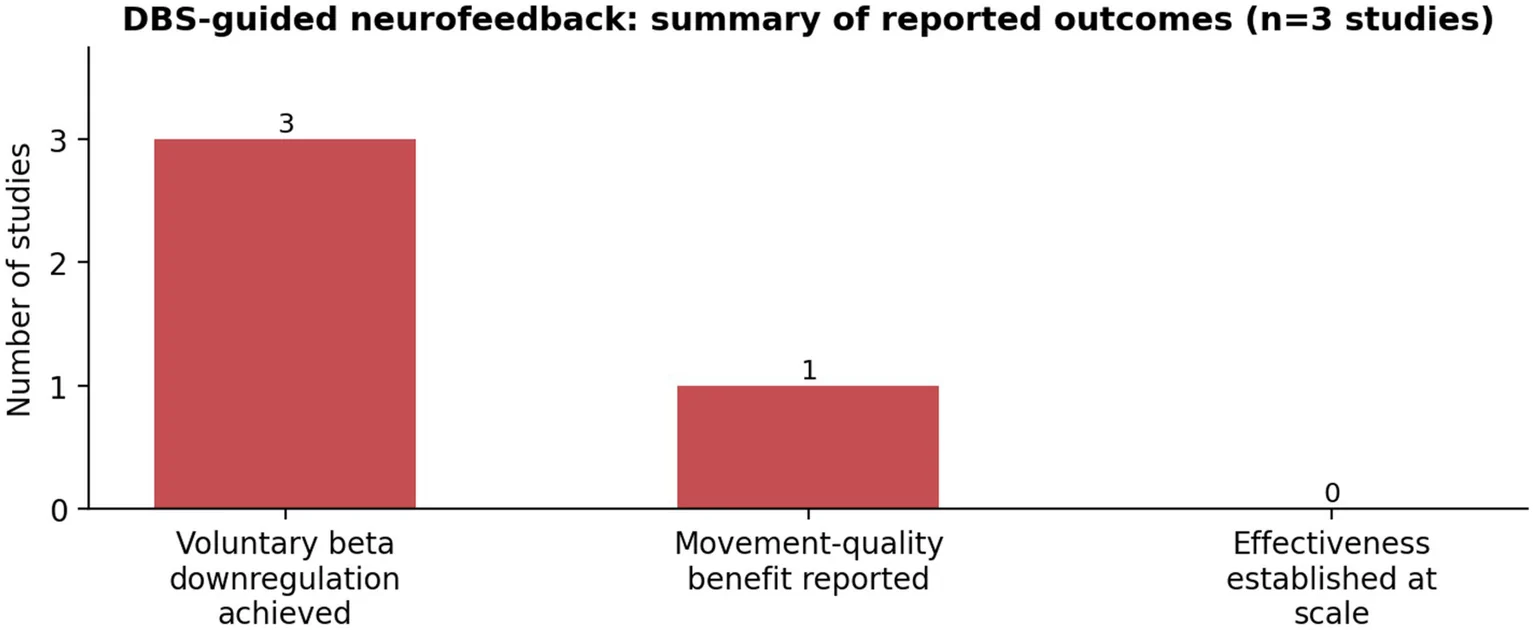
Summary of key DBS-guided neurofeedback results extracted from the studies in Table 2.

DBS-guided neurofeedback is one of the most biologically targeted NF strategies currently available in PD (22, 33). Its main strengths are pathophysiological specificity and direct access to disease-relevant neural signals. Its main limitations are equally clear: the approach is restricted to implanted patients, the evidence is still based on small and specialized cohorts, and clinical effectiveness has not yet been established at scale (22, 27, 33). Figure 4 summarizes, across the 3 DBS-guided neurofeedback studies reviewed above, how many reported successful voluntary beta downregulation, movement-quality benefit, and effectiveness established at scale.

## 4 Discussion

The present narrative review suggests that neurofeedback is a promising but still methodologically immature rehabilitation strategy for Parkinson’s disease. Across EEG-, fMRI-, and DBS-guided paradigms, the most consistent finding is that at least some patients with PD can learn to modulate disease-relevant neural activity, including cortical sensorimotor rhythms, movement-related cortical potentials, motor-network activity, and subthalamic beta oscillations (18, 20, 22, 31, 33). This is an important mechanistic result because it supports the biological plausibility of neurofeedback as a self-regulation-based intervention targeting dysfunctional neural circuits in PD (16, 17, 21).

Read chronologically rather than only by platform, the included studies also trace a discernible developmental trajectory. An initial phase (2002–2013) established basic proof of concept, largely through single-case or small-sample feasibility work showing that patients with PD could voluntarily influence a cortical signal at all, whether a sensorimotor rhythm, a movement-related cortical potential, or supplementary motor area activation (18, 29, 31, 36). A second phase (2014–2021) refined targeting and reporting, moving toward more structured EEG and fMRI protocols, exploratory behavioral outcomes, and the first proof-of-concept demonstration that subthalamic beta activity recorded from implanted DBS systems could itself be voluntarily modulated (17, 19, 30, 32). A third, more recent phase (2022–2026) is characterized by controlled or multi-arm comparisons, connectivity- and network-level fMRI targets, home-based and multimodal EEG protocols, and increasingly precise subcortical, implant-based targeting of beta-burst dynamics (20, 22–28, 37). Across this 24-year span, methodological sophistication and target specificity have clearly increased, yet sample sizes have grown only modestly and randomized designs remain the exception rather than the rule; the field has therefore matured considerably in terms of what can be feasibly targeted and measured, without a matching maturation in the evidence needed to establish clinical efficacy.

At the same time, the current literature clearly indicates that neurophysiological feasibility should not be conflated with clinical efficacy. In several studies, modulation of the intended neural target appears achievable, but downstream improvements in motor or non-motor outcomes are often modest, inconsistent, or limited to selected domains rather than generalized across overall disease severity (19, 24, 25, 27). In other words, successful signal control does not yet equate to robust therapeutic benefit. This distinction is central to the interpretation of the field and should guide future trial design (16, 21). The pattern recurs across all three platforms: EEG-NF studies most often demonstrate that the trained rhythm can be shifted with only partial and inconsistent motor or non-motor gain (24, 26); fMRI-NF studies show that regional activation or connectivity can be upregulated without a corresponding, reliably superior clinical effect relative to active control conditions (19, 20); and DBS-guided NF studies show that subthalamic beta power or beta-burst duration can be reduced on demand, while movement-quality benefit has so far been reported for only a subset of motor domains (27, 33). The consistency of this feasibility-efficacy gap across otherwise very different recording modalities suggests it reflects a genuine property of the field’s current evidence base rather than a limitation specific to any one platform.

Another major finding emerging from the available literature is the high degree of heterogeneity across studies. Existing reports differ substantially with respect to neurofeedback modality, neural target, feedback strategy, training intensity, number of sessions, medication state, control condition, and clinical outcome measures (16, 20, 21). Sample sizes are generally small, and many studies are case reports, pilot studies, or early translational investigations rather than adequately powered randomized trials (29, 33, 35, 36). Even among controlled studies, methodological variability limits direct comparison and weakens the possibility of determining whether one NF modality or protocol is superior to another (19, 23, 26). This heterogeneity is not only statistical but conceptual: studies differ in the sensory channel used to deliver feedback (visual, auditory, or combined), in whether training occurs in a single session or across multiple weeks, in whether patients are tested on or off dopaminergic medication, and in whether the comparator is a passive control, sham feedback, or an active alternative intervention. Because these design choices are rarely reported in a standardized way, it is currently difficult to determine which of them, if any, most strongly influences learning success or downstream clinical benefit. Notably, none of the 18 included studies specifically quantified event-related beta desynchronization or synchronization (ERD/ERS) as an outcome measure; most relied instead on absolute or relative beta-band power. Incorporating ERD/ERS metrics, which index the dynamic, movement-locked modulation of beta activity rather than its tonic level, could provide a more mechanistically sensitive marker of NF-induced change and represents a concrete methodological gap for future PD neurofeedback trials.

The available evidence also suggests that neurofeedback may be more clinically relevant as part of a multimodal rehabilitation framework than as a stand-alone intervention. This interpretation is supported by more recent studies in which combined approaches produced broader or stronger benefits than neurofeedback alone. For example, Romero et al. (26) reported that EEG-guided NF combined with bilateral high-frequency rTMS outperformed NF alone on several outcomes, suggesting that NF may act as a neural priming or self-regulation component that enhances the efficacy of other rehabilitative or neuromodulatory interventions. From a clinical perspective, NF may therefore be better conceptualized as an adjunctive strategy that complements physiotherapy, motor imagery, non-invasive stimulation, or device-based neuromodulation, rather than as an isolated treatment intended to replace established therapies (26, 38).

A further point concerns the differential translational value of the three main NF platforms. EEG-based neurofeedback remains the most accessible and scalable approach, given its lower cost, portability, and increasing adaptability to home-based training (21, 24). fMRI-based paradigms, while less practical for routine clinical deployment, continue to offer important mechanistic insights because they allow the targeting of specific cortical, subcortical, and connectivity-based motor networks, including the SMA and putamen (18, 20, 23). DBS-guided neurofeedback, in turn, offers perhaps the strongest pathophysiological specificity, since it directly engages pathological beta activity recorded from implanted therapeutic systems (17, 22, 33). However, this same specificity limits its applicability to selected implanted populations and to highly specialized research settings.

The translational outlook of the field remains encouraging, but greater ambition also introduces more demanding methodological requirements. Future studies should standardize the reporting of medication state, disease duration, disease stage, cognitive status, training adherence, and stimulation parameters where relevant, since all of these factors may influence both learning capacity and clinical response (16, 20, 21). Studies should also distinguish more clearly between three partially separable outcomes: successful modulation of the neural target, short-term behavioral change, and durable functional benefit. Without this distinction, there is a risk of overinterpreting proof-of-concept neurophysiological success as evidence of clinical readiness (19, 24, 33).

Longitudinal follow-up will also be essential. Much of the current literature evaluates immediate or short-term effects, but it remains unclear whether repeated neurofeedback can induce sustained neuroplastic changes that translate into stable functional gains over time (16, 21). This point is particularly important in PD, where disease progression, medication effects, and day-to-day symptom variability may all confound the interpretation of short training blocks or brief post-intervention assessments. In parallel, identifying predictors of responsiveness should become a major research priority, since it is increasingly plausible that NF will prove most useful only in selected subgroups with favorable cognitive, motivational, or neurophysiological profiles (20, 23).

The present evidence supports a cautious but positive interpretation. Neurofeedback in Parkinson’s disease appears biologically feasible, technically adaptable, and potentially relevant to both motor and selected non-motor domains (16, 21). Nevertheless, the field remains in an early translational phase, and the available studies do not yet justify routine clinical use. The next stage of research should therefore focus on adequately powered randomized trials, harmonized outcome measures, patient stratification strategies, longer follow-up periods, and better integration of neurofeedback with established rehabilitation and neuromodulation pathways (19, 21, 23, 26).

## 5 Challenges and future directions

Recent evidence suggests that the main challenge is no longer whether patients can modulate relevant neural signals, but whether such modulation translates into robust and reproducible clinical benefit. A 2025 meta-analysis indicates strong evidence for successful EEG target modulation, but low-certainty and inconclusive evidence for improvements in global motor symptom severity (21). This distinction is important for future trial design, as mechanistic success does not automatically translate into clinically meaningful change. Future studies should therefore include adequately powered randomized designs, standardized outcome sets, and longer follow-up intervals to determine whether repeated neurofeedback training produces durable functional gains (21).

Despite the promising results observed in early studies, the clinical application of neurofeedback (NF) in Parkinson’s disease (PD) faces several significant challenges that must be addressed before it can be widely implemented as a therapeutic intervention. One of the primary obstacles is the heterogeneity in NF protocols, as there is currently no standardized approach to training paradigms, frequency modulation targets, or session duration. Different studies utilize varied NF methodologies, feedback modalities, and reinforcement strategies, making it difficult to draw consistent conclusions regarding efficacy. Without standardization, comparing results across different studies remains a challenge, and the development of clinical guidelines for NF in PD is hindered. Establishing consensus on optimal NF parameters, session structure, and individualized training adjustments is crucial to advancing NF as a mainstream therapeutic option.

Another key limitation is the small sample sizes in NF studies, which restricts the generalizability of findings. Most NF research in PD has been conducted on small cohorts, often lacking sufficient statistical power to confirm long-term benefits or determine individual variability in response to treatment. Large-scale randomized controlled trials (RCTs) are essential to validate the effectiveness of NF across different stages of PD and to explore whether factors such as disease severity, comorbidities, and medication status influence NF outcomes. Longitudinal studies are needed to assess the durability of NF-induced neuroplastic changes and whether repeated training leads to sustained improvements in motor and cognitive functions over time.

Individual variability in NF learning capacity is another challenge that complicates widespread clinical application. Some PD patients demonstrate strong neurofeedback learning capabilities, successfully modulating their neural oscillations and experiencing functional improvements, while others struggle to engage effectively with NF training. This variability may stem from differences in cognitive flexibility, attentional capacity, neural plasticity, and motivation levels. Some PD patients exhibit reduced neurophysiological adaptability, particularly in advanced stages of the disease, which may limit their ability to benefit from NF. Another emerging issue concerns interindividual predictors of neurofeedback responsiveness. Recent translational work suggests that variability in clinical profile, disease stage, and cognitive characteristics may influence the capacity to modulate neural targets and the likelihood of translating such modulation into behavioral benefit. This reinforces the need for stratified and biomarker-informed approaches to patient selection in future PD neurofeedback trials (21, 39). Future research must explore predictors of NF responsiveness, including baseline EEG characteristics, cognitive profiles, and psychological factors, to identify which patients are most likely to benefit from NF interventions.

Another major challenge is the integration of NF with existing PD treatments, including pharmacological therapies, deep brain stimulation (DBS), and rehabilitative interventions. NF should not be viewed as a standalone treatment but rather as a complementary tool that can enhance the effectiveness of other therapies. For instance, NF may be used to optimize brain oscillatory patterns before or after DBS implantation or to reinforce motor training exercises used in physiotherapy. However, the interaction between NF and dopaminergic medication remains poorly understood, as dopaminergic treatments influence neural oscillations and may modulate NF responsiveness. Future studies should investigate whether NF can be tailored to synergize with pharmacological and neuromodulatory interventions to maximize therapeutic outcomes.

Looking ahead, the future of NF in PD rehabilitation is likely to be shaped by several technological innovations that could enhance accessibility, personalization, and effectiveness. Recent work supports the feasibility of home-based EEG neurofeedback (24), fully implanted DBS-based neurofeedback (33), and broader user-centered neurotechnology ecosystems for Parkinson’s disease rehabilitation and adaptive neuromodulation (39–44). These advances make it increasingly plausible that future neurofeedback interventions will be deployed in ecologically valid settings, but they also raise new demands regarding usability, safety, personalization, and integration with medication and device-based treatment strategies.

One of the most promising advancements is the development of AI-driven NF systems, which leverage machine learning algorithms to personalize NF training based on real-time brain activity. AI-driven NF could dynamically adjust feedback parameters, optimize reinforcement schedules, and detect patient-specific neural patterns to enhance training efficacy. By continuously adapting the training process to each individual’s neural responses, AI-enhanced NF systems could overcome some of the variability in learning capacity that currently limits NF’s clinical application.

The emergence of wearable NF devices represents another significant breakthrough in the field. Traditional NF training is often conducted in laboratory or clinical settings, limiting accessibility for many patients. However, the development of mobile EEG-based NF systems enables at-home rehabilitation, allowing PD patients to engage in self-paced, frequent training sessions without the need for constant clinical supervision. Portable NF devices could increase patient adherence and long-term engagement, making NF a scalable and cost-effective intervention. Integrating NF into consumer-friendly platforms, such as mobile applications and virtual reality interfaces, could further enhance usability and engagement.

Multimodal approaches that combine NF with other neuromodulatory and rehabilitation techniques hold great potential for enhancing therapeutic outcomes. Integrating NF with virtual reality (VR)-based motor rehabilitation could create an immersive training environment that strengthens motor learning and neuroplasticity. Biofeedback techniques, such as heart rate variability (HRV) training or electromyography (EMG) biofeedback, could be used alongside NF to enhance autonomic regulation and motor control. Combining NF with brain-computer interfaces (BCIs) may further enhance its clinical utility, allowing patients to translate NF-induced neural changes into functional motor commands for assistive technologies.

As neurofeedback technology continues to evolve, its role in PD rehabilitation is likely to expand beyond traditional EEG- or fMRI-based approaches. The integration of multi-modal neuroimaging techniques, closed-loop adaptive feedback systems, and neuroplasticity-enhancing interventions could transform NF into a powerful tool for precision medicine in neurodegenerative disorders. Future research should focus on optimizing NF training protocols, identifying biomarkers of NF responsiveness, and integrating NF with emerging digital health technologies to ensure its successful translation into routine clinical practice. While challenges remain, the rapid advancement of AI, wearable neurotechnology, and multimodal rehabilitation strategies suggests that NF could play a transformative role in the future of personalized PD therapy, offering a non-invasive, patient-driven approach to motor and cognitive enhancement.

## 6 Conclusion

In conclusion, neurofeedback represents an emerging and mechanistically grounded rehabilitation strategy for Parkinson’s disease. Current evidence indicates that patients with PD can, under specific conditions, learn to modulate targeted neural signals using EEG-, fMRI-, and DBS-guided paradigms, including cortical sensorimotor rhythms, supplementary motor area activity, connectivity-based motor networks, and subthalamic beta oscillations (18, 20, 22, 33). This supports the biological rationale of neurofeedback as an adjunctive neuromodulatory approach aimed at dysfunctional motor circuits and, potentially, selected non-motor domains (16, 21).

However, the current literature also indicates that neurophysiological feasibility is more strongly supported than clinical efficacy. Although preliminary studies report signals of benefit in selected motor and non-motor outcomes, these effects remain heterogeneous, often modest, and not yet generalizable across broader clinical endpoints or patient populations (19, 21, 24, 27). Neurofeedback should therefore be regarded, at present, as a promising adjunct rather than an established treatment for PD (16, 38).

Its future clinical value will depend on the development of standardized protocols, adequately powered randomized trials, clearer identification of responder profiles, and more effective integration with pharmacological, rehabilitative, and device-based therapies (21, 23, 26). If these challenges are addressed, neurofeedback may become a useful component of personalized neurorehabilitation in Parkinson’s disease, particularly as a strategy to complement rather than replace established treatments (17, 38).
